# Comparing suction rates of novel DuCanto catheter against Yankauer and standard suction catheter using liquids of different viscosity—a technical simulation

**DOI:** 10.1186/s12871-022-01830-2

**Published:** 2022-09-10

**Authors:** Simon-Richard Finke, Daniel C. Schroeder, Hannes Ecker, Bernd W. Böttiger, Holger Herff, Wolfgang A. Wetsch

**Affiliations:** 1 Department of Anesthesiology and Intensive Care Medicine, University Hospital of Cologne, Kerpener Str. 62, 50937 Cologne, Germany; 2 Faculty of Medicine, University of Cologne, 50937 Cologne, Germany; 3 Department of Anesthesiology and Intensive Care, German Armed Forces Central Hospital Koblenz, Koblenz, Germany; 4 Department of Anesthesiology, PAN Clinic, Cologne, Germany

**Keywords:** Airway, Aspiration, DuCanto, Mendelson syndrome, Suctioning catheter, Regurgitation, Yankauer catheter

## Abstract

**Purpose:**

Aspiration is a feared complication that may occur during airway management, and can significantly contribute to morbidity and mortality. Availability of a suctioning device with a suction catheter capable of clearing the airway is mandatory for airway management. However, suction performance may be significantly different amongst different suction catheters. The aim of this study was to compare suction rates of a standard 14 Ch suction catheter (SC), a Yankauer catheter (Y) and a DuCanto catheter (DC) using 4 fluids with different viscosity.

**Methods:**

In this simulation trial, 4 preparations with standardized viscosity were prepared using a Xanthane-based medical fluid thickener. Lowest viscosity was achieved using tap water without thickener, syrup-like viscosity was achieved by adding 10 g per liter tap water, honey-like viscosity was achieved by adding 20 g per liter, and a pudding-like viscosity was achieved by adding 30 g of thickening powder per liter tap water. Each preparation was suctioned for 15 s with the three different suctioning devices. Measurements were repeated four times. The amount of removed preparation by suctioning was measured using a tared scale.

**Results:**

Suction rates for water were 580 ± 34 mg for SC, 888 ± 5 mg for Y and 1087 ± 15 for DC; for syrup-like viscosity it was 383 ± 34(SC) vs. 661 ± 64(Y) vs. 935 ± 42(DC); for honey-like viscosity it was 191 ± 21(SC) vs. 426 ± 34(Y) vs. 590 ± 68(DC); and for pudding-like viscosity 74 ± 13(SC) vs. 164 ± 6(Y) vs. 211 ± 8(DC).

**Conclusion:**

Suctioning liquids of different viscosity, the new DuCanto catheter was more effective than the Yankauer catheter that was more effective than a standard suctioning catheter. The relative superiority of the DuCanto was highest in fluids with high viscosity.

## Introduction

Failed airway management remains a major contributor towards morbidity and mortality in modern anesthesia, intensive care and emergency medicine [1–3]. The knowledge of the importance of a secure airway for general anesthesia is almost as old as modern anesthesia itself. Following the first public demonstration of general anesthesia with ether by William Thomas Morton in 1846, James Young Simpson reported the first death due to pulmonary aspiration during chloroform induced anesthesia in 1848 [4]. Today, anesthesiologists, critical care and emergency doctors take multiple precautions in order to prevent pulmonary aspiration when inducing general anesthesia, but regurgitation of gastric content can occur despite all precautions, as a review of 166,491 anesthesia records in a recent study showed: in 20 cases, they found pulmonary aspiration, and in an additional 20 cases there were signs of regurgitation without pulmonary aspiration [5]. Pulmonary aspiration can have varying clinical consequences – ranging from clinically none, to aspiration pneumonia due to bacteria transported into the lung alongside with the aspirate, [6] right up to severe acute respiratory distress syndrome (ARDS) with distinct hypoxemia – named “Mendelson Syndrome" after Curtis L. Mendelson, who described a case series of 66 healthy obstetrical patients with chemical pneumonitis after ether anesthesia [7]. Whenever a patient is at risk for aspiration, a modified intubation technique called “rapid sequence induction” (RSI) is accepted as the technique of choice for securing the airway [8]. When performing RSI, induction agents are injected in rapid sequence and without bag-mask ventilation [9]. Usually, a large-bore suction catheter will be available to suction any aspirate that might occur during the procedure. These suction catheters may have difficulties as suction rates can decline with increasing viscosity of the aspirate and may be blocked by particles within the aspirate [10]. However, there are different possibilities of what kind of fluid must be suctioned to clear the airway – from watery solutions such as beverages, to stomach acid, airway secretions, blood, and coagulated blood, and different kinds of food. Accordingly, different levels of viscosity must be expected and handled.

To our knowledge. The DuCanto catheter represent the largest-bore commercially available suction catheter for adult patients. Therefore, in this study, we evaluated the DuCanto catheter against our current clinical standard Yankauer suction catheter, and the largest clinically available standard suction catheter, using liquids of four standardized viscosities in a prospective technical simulation study. Primary endpoint was suction rate (in mg/min) of the different catheters with different standardized viscosities. Our formal hypothesis was that there are no differences between groups.

## Materials and methods

### Ethics approval

This study was designed as a technical simulation and was conducted in our laboratory at the University Hospital of Cologne. As neither patients nor patient data were investigated, approval from the local Ethics Committee was not required for this type of study.

### Experimental setup

**Fig. 1:**
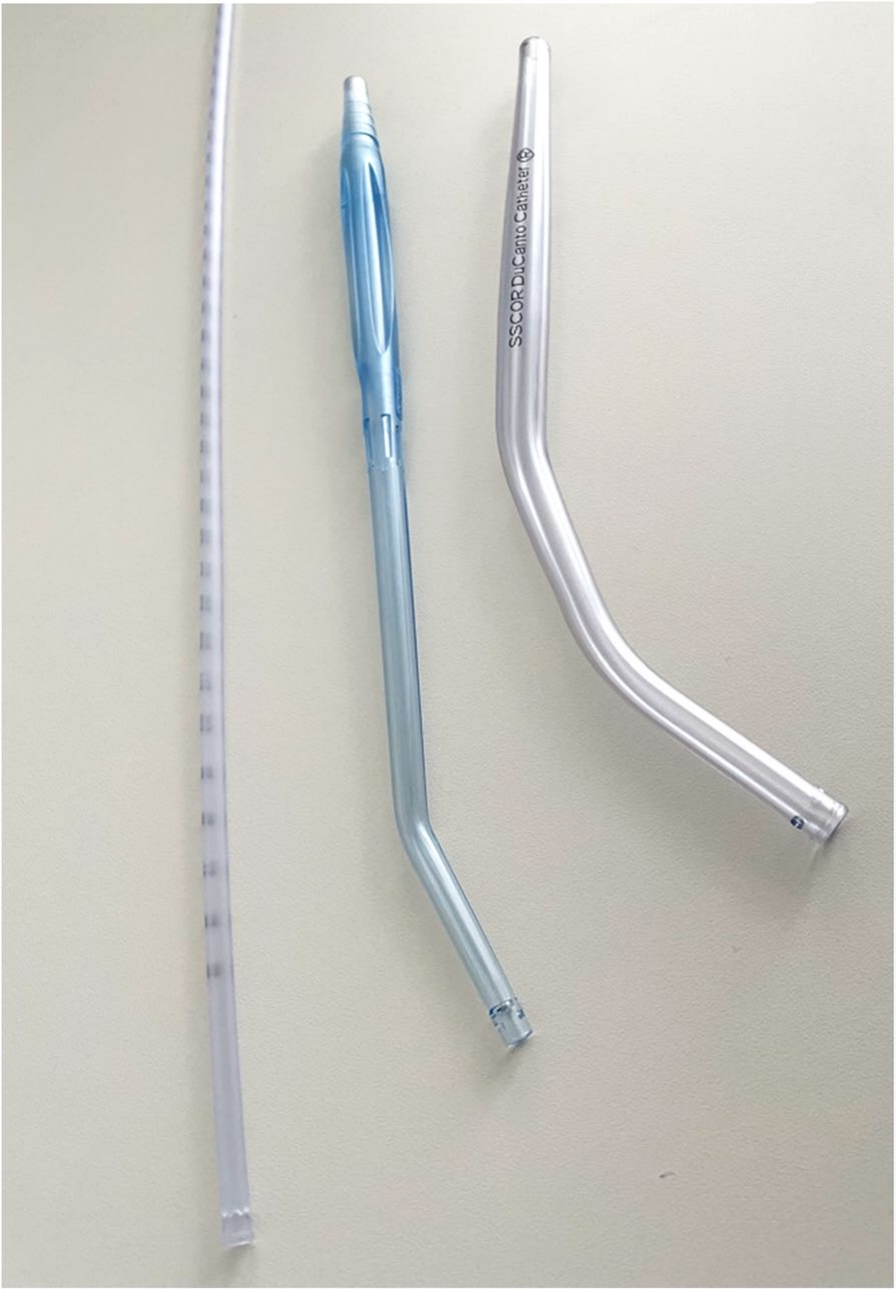
ConvaTec 14Ch suction catheter (left), Extrudan Yankauer 18Ch suction catheter (middle) and SSCOR DuCanto 28Ch suction catheter (right)

We compared (a) a standard 14 Charrière (Ch) suction catheter (ProFlo with funnel, outer diameter 4.667 mm [Ch 14], length 60 cm; ConvaTec Ltd., Deeside, United Kingdom), (b) a standard large-bore rigid Yankauer suction catheter (Yankauer Midi, outer diameter 6.0 mm [Ch 18], length 27 cm; EXTRUDAN Surgery ApS, Birkerød, Denmark), both of which were in use in clinical daily routine, and (c) the novel SSCOR DuCanto Catheter (DuCanto Catheter with outer diameter 9.3 mm [Ch 28], length 24.9 cm; SSCOR, Inc., Sun Valley, CA, United States of America). Figure 1 shows the suction catheters used. These were connected to a standard suction line used for clinical routine in our hospital (suction line 1.8 m, Ch 25; Ref. 1324.1800.80ML, Extrudan Surgery ApS, Denmark). Negative pressure of the suction unit was set to the maximum, which was -0.8 bar (approx.—600 mmHg).

We measured the suction rates of each catheter using four different fluids with standardized levels of viscosity. To achieve these, a Xanthan-based thickening medium for use in patients with dysphagia (seneoPro VISCOclear instant, biozoon GmbH, Bremerhaven, Germany) was added to tap water to achieve standardized viscosities, as instructed by the manufacturer [11]. Lowest viscosity (A) was achieved using tap water without thickener, syrup-like viscosity (B) was achieved by adding 10 g per liter tap water, honey-like viscosity (C) was achieved by adding 20 g per liter, and a pudding-like viscosity (D) was achieved by adding 30 g of thickening powder per liter tap water. These mixtures were stirred well using a whisk to ensure a homogenously distribution, and thickening was allowed for 5 min prior to starting to ensure that a proper effect had taken place according to the manufacturer’s specifications.

### Experimental procedure

Measurements were repeated four times for each suction catheter. Suctioning was accomplished by keeping the tip of every catheter beneath the surface, but markedly above the bottom of the container to avoid measuring errors by inlet of air, or with the bottom of the container that might reduce the actual suction rate. Suctioning of the respective fluid with the respective catheter was executed for exactly 15 s each, measured using a laboratory timer (Timer 38.2010, TFA Dostmann GmbH & Co. KG, Wertheim-Reicholzheim, Germany). After each attempt, the suctioning bag was removed, the scale was tared using the tare function to the weight of an empty suctioning bag, and the full suctioning bag was weighted by means of a calibrated, gauged infant scale (seca 376, seca GmbH & Co. KG, Hamburg, Germany). Suction rates were calculated accordingly. The primary outcome of this study was the weight measured by full grams of mobilized test fluid in a 15 s timeframe.

### Statistical analysis

Data was analyzed using Sigmaplot 14 (Systat, San Jose, CA). Data was tested for normal distribution using Shapiro–Wilk test, and equal variances were evaluated with Brown-Forsythe test. If given, a one-way analysis of variance (ANOVA) was used to determine statistically significant differences. Finally, an all pairwise multiple comparison was made using Holm-Sidak method. Figures were created using Prism 9 (GraphPad, San Diego, CA, USA).

## Results

**Fig. 2:**
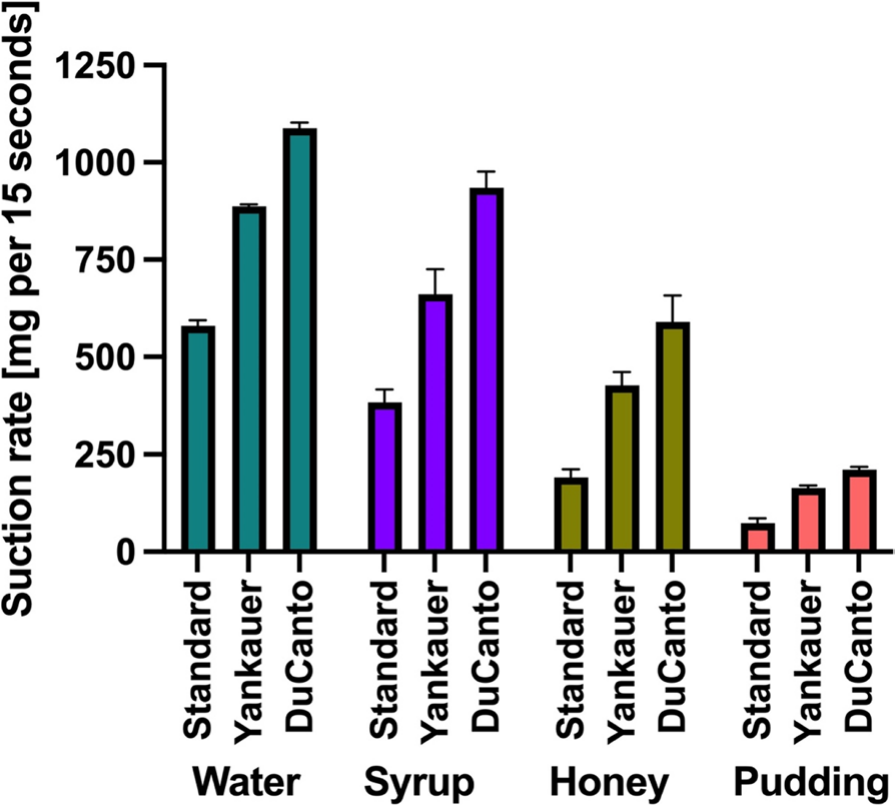
Visualization of mean values of suction performance per 15 s using the three different suction catheters at fluids of four different viscosities

The suction rates achieved by the suction catheters and the different viscosities are shown in Table 1, and a visualization of the suction performance can be found in Fig. 2.

**Table 1:** Mean values and standard deviations (SD) of the different suction catheters and the different viscosities

	**Suction catheter (a)**			**Yankauer (b)**			**DuCanto (c)**		
	n	mean weight	SD	n	mean weight	SD	n	mean weight	SD
**Water (A)**	4	580.0	14,1	4	887.5	5.0	4	1087.5	15.0
**Syrup (B)**	4	382.5	34.0	4	661.3	64.1	4	935.0	42.0
**Honey (C)**	4	191.3	21.0	4	426.3	34.2	4	590.0	68.1
**Pudding (D)**	4	73.8	12.5	4	163.8	6.3	4	211.3	7.5

For water, after testing for normality with Shapiro–Wilk test and equal variances with Brown-Forsythe test, one-way analysis of variance (ANOVA) revealed a statistically significant difference (*p* < 0.001). An all pairwise multiple comparison (Holm-Sidak method) revealed significant differences between each suction catheter (Table 2).

**Table 2:** Comparison of the performance of the different suction catheters with water, syrup, honey, and pudding

**Medium**	**Suction Catheter**	**Difference of means**	**t**	***P***
**Water**	DuCanto vs. standard	507.5	58.601	< 0.001
	Yankauer vs. standard	307.5	35.507	< 0.001
	DuCanto vs. Yankauer	200.0	23.094	< 0.001
**Syrup**	DuCanto vs. standard	552.5	16.140	< 0.001
	Yankauer vs. standard	278.8	8.143	< 0.001
	DuCanto vs. Yankauer	273.8	7.997	< 0.001
**Honey**	DuCanto vs. standard	372.5	8.799	< 0.001
	Yankauer vs. standard	235.0	5.551	< 0.001
	DuCanto vs. Yankauer	137.5	3.248	0.001
**Pudding**	DuCanto vs. standard	137.5	21.213	< 0.001
	Yankauer vs. standard	90.0	13.885	< 0.001
	DuCanto vs. Yankauer	47.5	7.328	< 0.001

For syrup, after passed Shapiro–Wilk and Brown-Forsythe testing for normality and equal variances, one-way ANOVA revealed a significant difference. All pairwise comparisons (Holm-Sidak test) showed significant differences between each suction catheter (Table 2).

For honey, after testing for normality (Shapiro–Wilk) and equal variance (Brown-Forsythe), one-way ANOVA showed significant difference. All pairwise comparisons (Holm-Sidak test) showed significant differences between each suction catheter (Table 2).

For pudding, normality was again verified with Shapiro–Wilk and equal variance with Brown-Forsythe tests, and one-way ANOVA showed a significant difference. All pairwise comparisons (Holm-Sidak test) showed significant differences between all suction catheters (Table 2).

## Discussion

In this study, we evaluated the suction rates of a standard suction catheter, a large-bore Yankauer suction catheter, and the new large-bore DuCanto suction catheter, when using fluids with four different standardized degrees of viscosity in a technical simulation. The results of this study showed that large-bore catheters were able to achieve significantly higher suction rates in all degrees of viscosity.

Failed airway management due to gastric regurgitation or hemorrhage and subsequent impeded visibility is a dreaded situation in anesthesia, critical care and emergency medicine [12]. Every second of poor visibility and obstructed airway control may have incremental effects in patient health and lead to hypoxemia or death [13]. The importance of a fully functioning, ready to use high performance suction device is further recommended to be incorporated into every workplace that provides elective and emergency airway management [14].

There are several studies and case reports that compared different sizes of the Yankauer with different sizes of other suction devices (including endotracheal tubes connected to a meconium aspirator) [15–18]. In these studies, a lager diameter of the suction device improved the suction performance, as it is intuitively expected, and be explainable by the law of Hagen and Poiseulle. Interestingly, in this study the difference between the standard catheter and the DuCanto is less than twice the suctioning rate for water, whereas for the pudding like consistence it is approximately three times higher, which can be explained by more turbulent flows in catheters with smaller diameters and increasing viscosity.

It is noteworthy, that a suctioning rate of rounded 74 mg per 15 s with a standard catheter, even an amount of regurgitated stomach contents of only 250 mL will take nearly one minute to be completely removed; this is a very long time in an emergency situation with regurgitation, aspiration and most probably in consequence deteriorating arterial oxygenation [19]. The largest-bore of the catheters we compared would need only 17.5 s for the same amount with the same viscosity.

Further, the longer it takes to remove the regurgitated stomach contents, the higher the likelihood is that massive aspiration occurs, which may result in a severe or even deadly Mendelson syndrome [20]. We deliberately did not use preparations with corpuscular element since these cannot be standardized. One may imagine that every meal has a different consistence, depending on eating behavior, dental status, contact time with digestive enzymes and gastric acidity. However, depending on the latter, it is most likely that normal stomach contents include corpuscular elements, which may further have the potential to deteriorate suction rate or even obstruct suction catheters with lower diameters. So, our values may be considered as maximum rates when no corpuscular elements are present, and can be expected much lower in real-life situations.

The Yankauer catheter is one of the most common suction devices and has become the standard tool used by emergency physicians during endotracheal intubation [15]. In this regard, the new DuCanto Catheter with its 50% better performance in suctioning rate in high viscosity preparations seems to be a very promising alternative.

We have chosen to use four different levels of viscosity to simulate fluids that can typically occur in clinical situation (water as a reference, syrup for stomach acid, honey for airway secretes, and pudding for freshly coagulated blood). Of course, our mixture of xanthan-based thickener and water only gives a surrogate of how these could be, but on the other side, in real life, coagulated blood or stomach acid are not standardized either. This is why we chose to use this product to ensure reproducibility of our experiments. As for the suction unit, we have chosen -600 mbar to represent the maximum suction rate that is available at all work places of a huge university hospital, to simulate the situation that would occur in the clinical setting.

We face several limitations in this study. First, a bench model study is always a limitation by itself, e.g. it can never simulate the stressful situation of a real emergency situation. However, on the other side it is nearly if not totally impossible to study aspirations in prospective controlled clinical trials. Observational studies need large numbers of observed patients and can never be standardized. Thus, we are confident our model is a reproduceable and valid method to assess function of suction devices, such as the new DuCanto Catheter. Secondly, the study staff could not be blinded to the catheter they used. Third, as mentioned above we did not use corpuscular stomach contents, which would be different in humans. Last, we did not test these catheters in an intubation scenario that may be able to demonstrate usefulness of the DuCanto for this standard anesthesia procedures [21–23].

In conclusion, suctioning substances of different viscosity from the upper airway the new DuCanto catheter was more effective than the Yankauer catheter, which was more effective compared to a standard suctioning catheter. The relative superiority of the DuCanto was highest in fluids of high viscosity.

## Data Availability

The original datasets analysed during the current study are available from the corresponding author on reasonable request.
